# Population-wide persistent hemostatic changes after vaccination with ChAdOx1-S

**DOI:** 10.3389/fcvm.2022.966028

**Published:** 2022-07-29

**Authors:** Bas de Laat, Hendrik Stragier, Romy de Laat-Kremers, Marisa Ninivaggi, Dieter Mesotten, Steven Thiessen, Kristien Van Pelt, Mark Roest, Joris Penders, Pascal Vanelderen, Dana Huskens, Raf De Jongh, Margot Vander Laenen, Tom Fivez, Hugo ten Cate, Rene Heylen, Line Heylen, Deborah Steensels

**Affiliations:** ^1^Department of Functional Coagulation, Synapse Research Institute, Maastricht, Netherlands; ^2^Department of Data Analysis and Artificial Intelligence, Synapse Research Institute, Maastricht, Netherlands; ^3^Department of Anesthesiology, Intensive Care Medicine, Emergency Medicine and Pain Therapy, Hospital Oost-Limburg, Genk, Belgium; ^4^CARIM School for Cardiovascular Diseases, Faculty of Health, Medicine and Life Sciences, Maastricht University, Maastricht, Netherlands; ^5^UHasselt, Faculty of Medicine and Life Sciences, Diepenbeek, Belgium; ^6^Department of Laboratory Medicine, Ziekenhuis Oost-Limburg, Genk, Belgium; ^7^Department of Platelet Pathophysiology, Synapse Research Institute, Maastricht, Netherlands; ^8^Thrombosis Expertise Center, Department of Internal Medicine, Maastricht University Medical Center, Maastricht, Netherlands; ^9^Department of Cardiovascular Sciences, Section Anesthesiology and Algology KULeuven, Leuven, Belgium; ^10^Department of Nephrology, Ziekenhuis Oost-Limburg, Genk, Belgium; ^11^Université Libre de Bruxelles, Faculty of Medicine, Brussels, Belgium

**Keywords:** ChAdOx1-S, COVID-19, thrombin generation, vaccination, hemostasis

## Abstract

Various vaccines were developed to reduce the spread of the Severe Acute Respiratory Syndrome Cov-2 (SARS-CoV-2) virus. Quickly after the start of vaccination, reports emerged that anti-SARS-CoV-2 vaccines, including ChAdOx1-S, could be associated with an increased risk of thrombosis. We investigated the hemostatic changes after ChAdOx1-S vaccination in 631 health care workers. Blood samples were collected 32 days on average after the second ChAdOx1-S vaccination, to evaluate hemostatic markers such as D-dimer, fibrinogen, α2-macroglobulin, FVIII and thrombin generation. Endothelial function was assessed by measuring Von Willebrand Factor (VWF) and active VWF. IL-6 and IL-10 were measured to study the activation of the immune system. Additionally, SARS-CoV-2 anti-nucleoside and anti-spike protein antibody titers were determined. Prothrombin and fibrinogen levels were significantly reduced after vaccination (−7.5% and −16.9%, *p* < 0.0001). Significantly more vaccinated subjects were outside the normal range compared to controls for prothrombin (42.1% vs. 26.4%, *p* = 0.026) and antithrombin (23.9% vs. 3.6%, *p* = 0.0010). Thrombin generation indicated a more procoagulant profile, characterized by a significantly shortened lag time (−11.3%, *p* < 0.0001) and time-to-peak (−13.0% and *p* < 0.0001) and an increased peak height (32.6%, *p* = 0.0015) in vaccinated subjects compared to unvaccinated controls. Increased VWF (+39.5%, *p* < 0.0001) and active VWF levels (+24.1 %, *p* < 0.0001) pointed toward endothelial activation, and IL-10 levels were significantly increased (9.29 pg/mL vs. 2.43 pg/mL, *p* = 0.032). The persistent increase of IL-10 indicates that the immune system remains active after ChAdOx1-S vaccination. This could trigger a pathophysiological mechanism causing an increased thrombin generation profile and vascular endothelial activation, which could subsequently result in and increased risk of thrombotic events.

## Introduction

At the end of 2019, the Severe Acute Respiratory Syndrome-Cov-2 (SARS-CoV-2) virus caused a pandemic that has been keeping the world in its grip ever since. By December 2021, more than 260 million people were infected with the virus and over 5.3 million people died as a result of the SARS-CoV-2 virus (1, 2). Several vaccines were developed to reduce the spread of the SARS-CoV-2 virus, including the ChAdOx1-S vaccine, formerly known as “COVID-19 Vaccine AstraZeneca” (3, 4). The ChAdOx1-S vaccine leads to the synthesis of specific SARS-CoV-2-proteins to elicit an immune response against the spike protein of SARS-CoV-2. The ChAdOx1-S vaccine uses a modified chimpanzee-derived adenovirus encoding for the spike-glycoprotein ChAdOx1-S of SARS-CoV-2 (5). Since January 2021, the ChAdOx1-S vaccine has been administered worldwide but predominantly in the UK (6). A study by Jeong et al. (7) reported a SARS-CoV-2 antibody positivity rate of 98.2–100% after two vaccinations, and the overall estimated vaccine efficacy was 74.0% (3). Quickly after the start of vaccination, reports started to emerge that anti-SARS-CoV-2 vaccines, including ChAdOx1-S, could be associated with an increased risk of thrombotic events (5).

In April 2021, European Medicines Agency reported a possible association between ChAdOx1-S and other SARS-CoV-2 vaccines, and a rare syndrome of thrombosis alongside thrombocytopenia, which was named vaccine-induced thrombotic thrombocytopenia (VITT) (8). VITT presents as thrombosis in atypical sites, thrombocytopenia and the presence of autoantibodies against platelet-factor 4 (PF4) (9). The autoantibody against PF4 binds to the platelet FcRγIIA receptor and causes platelets to aggregate.

As vaccination with ChAdOx1-S as well as with Ad26.COV2.S has been linked not only to VITT, but also to other venous and arterial thrombotic episodes without thrombocytopenia (10), we were interested whether vaccination with ChAdOx1-S would be associated with more global hemostatic changes post-vaccination. Therefore, we studied a large range of hemostatic parameters in a cohort of health care workers in a Belgian tertiary center, Ziekenhuis Oost Limburg (ZOL), 4 weeks after the second dose of the ChAdOx1-S vaccine.

## Methods

### Participants and sample handling

Health care workers who received the second dose of the SARS-CoV-2 vaccine ChAdOx1-S at the Hospital Oost Limburg (ZOL) hospital, a tertiary center in Belgium, were invited for blood sampling. The study was in accordance with the declaration of Helsinki, and approved by the local medical ethics committee and by the federal agency for medicines and health products (FAMHP). All participants gave written informed consent. Subjects with known coagulation defects or subjects below 18 years of age were excluded from the study.

Control samples were collected prior to the start of the COVID-19 pandemic after approval by the local medical ethics committee of the Maastricht University Medical Center and after written informed consent. Subjects with known coagulation defects, subjects below 18 years of age or individuals using anticoagulants were excluded from the study. As the vaccinated health care worker cohort consists primarily of women, the control group consisted of subjects that were matched for sex and age. Additionally, reference ranges for each parameter were determined in a group of 120 healthy individuals, consisting of 50% men, 37% women without oral contraceptives, and 13% women with oral contraceptives.

Blood was collected on 0.109 M sodium citrate (9 NC coagulation sodium citrate 3.2%, Greiner Bio-One GmbH, Austria) for analysis of hemostatic parameters. Serum (BD vacutainer SST II Advance, BD, UK) was prepared for the detection of anti-SARS-CoV-2 antibodies. Citrated blood was centrifugated twice at 2,821 g for 10 min to prepare platelet poor plasma. Serum tubes were centrifugated once at 2,821 g for 10 min to prepare serum. All samples were aliquoted and stored at −80°C until further use.

### COVID-19 related tests

**Figure 1:**
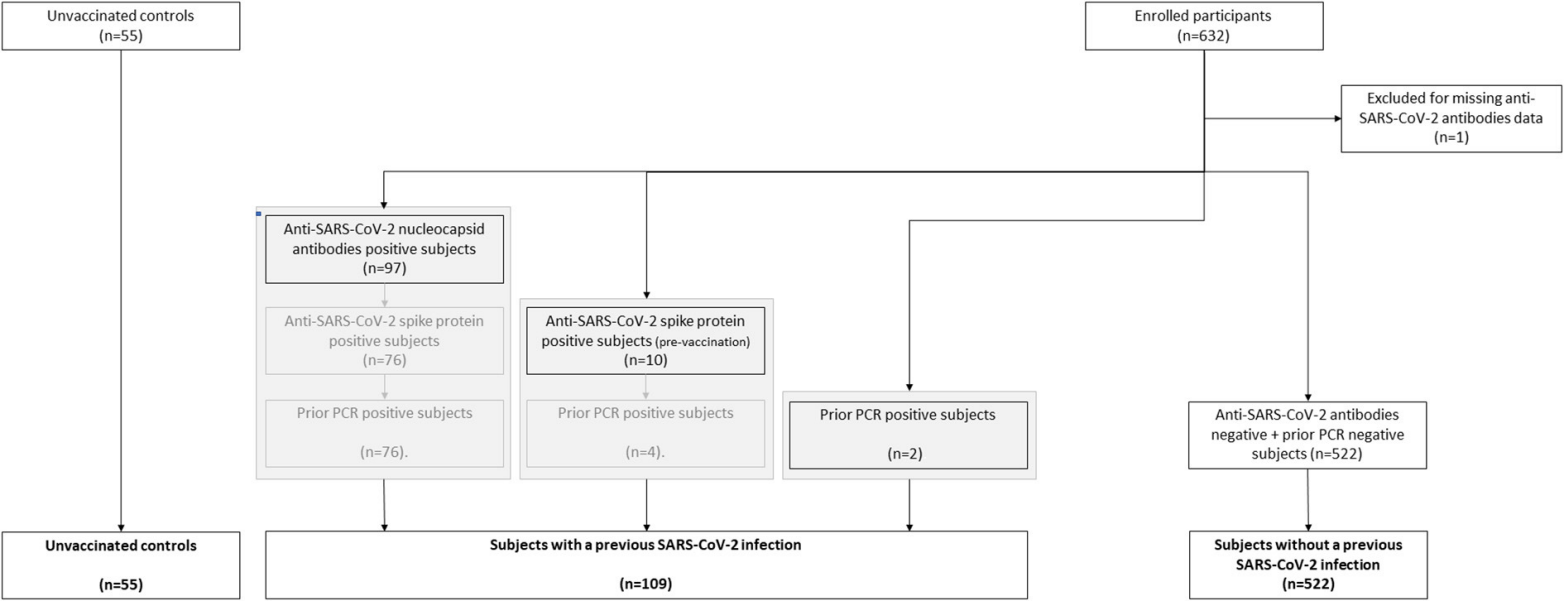
Flow chart of the classification of subjects vaccinated with ChAdOx1-S with and without prior COVID-19.

SARS-CoV-2 anti-S antibodies were measured using the quantitative Elecsys anti-RBD, in addition to semi-quantitative Elecsys anti-N (both measuring total immunoglobulin levels) on the cobas e801 analyzer (Roche Diagnostics, Rotkreuz, Switzerland). Results for the quantitative Elecsys anti-RBD antibodies are reported as concentrations (U/mL), with a manufacturer's cut-off > 0.8 U/mL considered as positive. Results for the Elecsys anti-N antibodies are reported as a cut-off index (signal sample/cut-off or signal calibrator), with values > 1 considered as positive. A previous history of COVID-19 could be established by the presence of anti-SARS-CoV-2 nucleocapsid antibodies (post-vaccination), the presence of anti-SARS-CoV-2 spike protein antibodies (pre-vaccination), a positive PCR test (pre-vaccination) or any combination (Figure 1).

### Hemostatic parameters and coagulation factors

D-dimer, fibrinogen, antithrombin and FVIII were measured on the STA-R (Diagnostics Stago, Asnières, France), using STA-Liatest D-di Plus, STA-Liquid Fib, STA-Stachrom AT III and STA-Immunodef FVIII reagents (Diagnostics Stago, Asnières, France), respectively according to the manufacturer's specifications. Von Willebrand Factor (VWF) and active VWF levels were measured with an in house ELISA, as described in detail elsewhere (11). Functional α_2_M levels were measured with an in-house method as previously described (12).

### Thrombin generation

Thrombin generation (TG) was measured in platelet poor plasma by the calibrated automated thrombinography (CAT) method, using PPP reagent, PPP reagent low, Calibrator and FluCa kits (Diagnostics Stago, Asnières, France) according to the manufacturer's specifications. TG was measured in the presence or absence of thrombomodulin (TM; 50% inhibition of the peak height in pooled normal plasma) to test the sensitivity of the activated protein C (APC) system. TG curves and parameters were generated by the dedicated CAT software.

### Measurement of interleukins

IL-6 and IL-10 concentrations were determined by ELISA using the BMS213 kit (Invitrogen, Waltham, Massachusetts) and BMS215 kit (Invitrogen, Waltham, Massachusetts), respectively, in accordance with the manufacturer's instructions.

### Clinical adverse effects

All health care workers in Hospital Oost-Limburg were invited to report adverse effects after SARS-CoV2 vaccination through a questionnaire at day 8 after each vaccine dose. Clinical symptoms were reported as none, mild, moderate or severe for each symptom, except insomnia, which was classified as none, mild or moderate, and anaphylaxis and facial paralysis, which was reported as present or not present. For further analysis, clinical symptoms were either classified as “injection site symptoms” (tenderness of the arm and redness of the arm), or “systemic symptoms” (fatigue, headache, muscle pain, chills, fever, joint pain, insomnia, nausea, swollen lymph nodes, facial paralysis, and anaphylaxis).

### Statistical analyses

Differences between groups were determined using the Chi^2^ test for categorical data and ANOVA or Kruskal-Wallis analysis for continuous data, depending on the distribution of the data. When comparing two groups, either the *t*-test or the Mann Whitney test was used instead. Statistical significance is indicated as ^*^, ^**^, and ^***^, respectively for *p*-values below 0.05, 0.01, and 0.001. Statistical analyses were performed in GraphPad Prism (version 8; GraphPad Software, San Diego, California), and IBM Statistical Package for Social Sciences (SPSS; version 25; SPSS Incorporated, Chicago, Illinois).

## Results

We assessed the hemostatic profile in 631 health care workers vaccinated with SARS-CoV-2 vaccine ChAdOx1-S and in 55 controls (Table 1). The majority of the vaccinated population was female (85.4%) and the median age was 41 years. Age and sex were comparable in the control group, which consisted of 51 women (93%) with an average age of 39 years. In the vaccinated subjects, blood sampling was performed on average 32 days (range 21–40 days) after the second dose of ChAdOx1-S. All vaccinated participants developed anti-SARS-CoV-2 spike protein antibodies. Anti-SARS-CoV-2 nucleocapsid antibodies, related to a previous COVID-19 infection, were present in 13.9% of the vaccinated subjects. A total of 109 participants (17.3%) suffered from COVID-19 in the past (Figure 1). In this subset of individuals, in which COVID-19 infection was confirmed during the infection with a PCR test, the median time between the COVID-19 infection and the blood drawing is 34 weeks (IQR 26–60 weeks). Furthermore, clinical symptoms were recorded during the study, and no thrombotic or hemorrhagic complication was reported after vaccination.

**Table 1:** General characteristics of controls subjects and subjects vaccinated with ChAdOx1-S.

	**Control subjects**	**Vaccinated subjects without previous SARS-CoV-2 infection**	**Vaccinated subjects with previous SARS-CoV-2 infection**	***P*** **-value**
*N*	55	522	109	
Age, years (SD)	39 (9)	40 (9)	39 (10)	n.s.
Sex, number of men (%)	4 (7%)	81 (15.5%)	11 (10.0%)	n.s.

Data are displayed as mean and standard deviation or as percentages in the case of categorical variables. Differences between the groups were analyzed by ANOVA. A p-value below 0.05 was considered statistically significant.

### ChAdOx1-S vaccination and procoagulant factor levels

**Figure 2:**
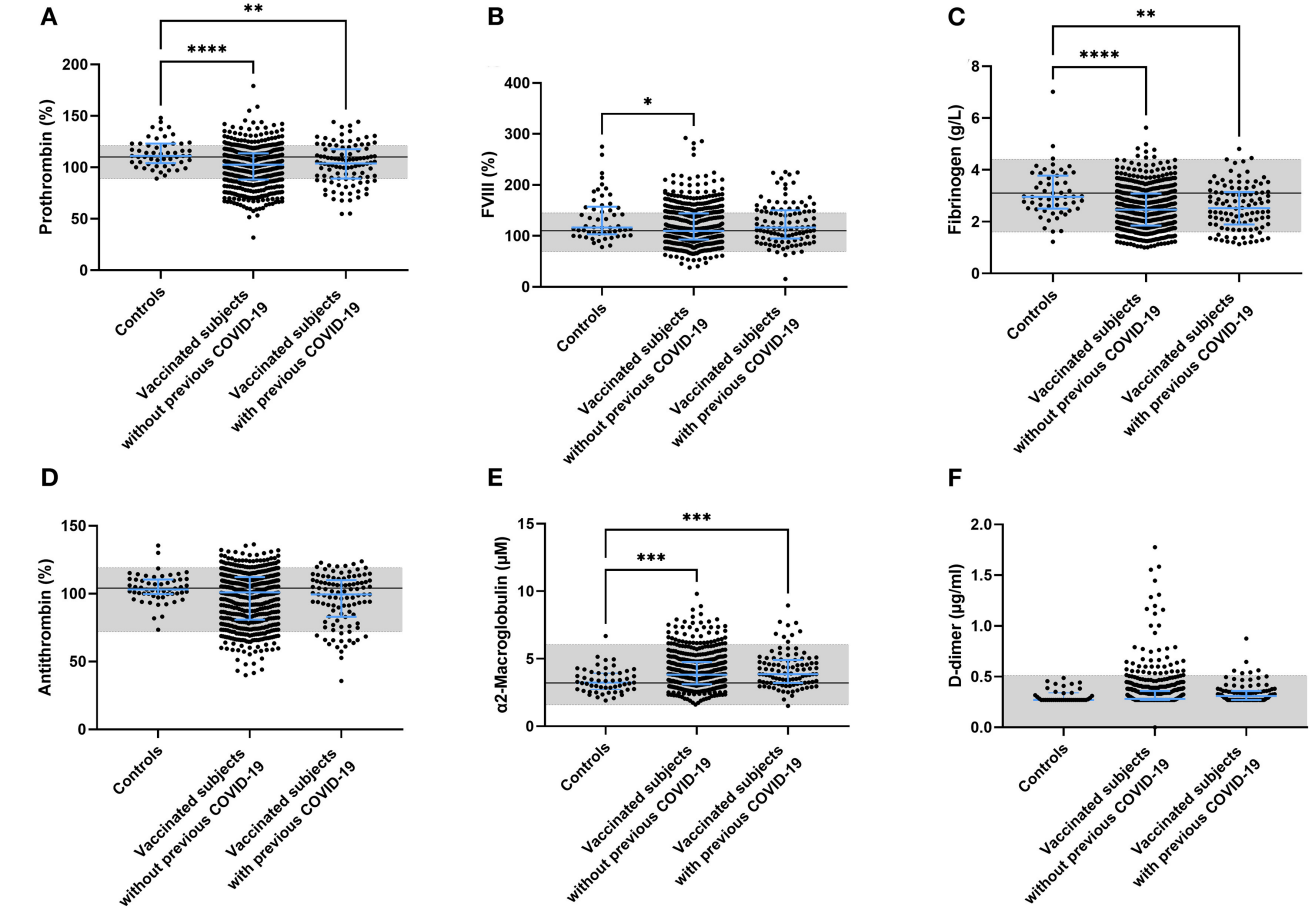
Coagulation factor levels in controls and subjects vaccinated with ChAdOx1-S with and without prior COVID-19 infection. Plasma levels of prothrombin **(A)**, FVIII **(B)**, fibrinogen **(C)**, antithrombin **(D)**, α2-macroglobulin **(E)** and D-dimer **(F)** were quantified. Reference values for each test are indicated as the gray area in the dot plot. Differences between the groups were analyzed by ANOVA or Kruskal-Wallis analysis, depending on the distribution of the data. *P*-values below 0.05, 0.01, 0.001, and 0.0001 were marked as ^*^, ^**^, ^***^, and ^****^, respectively.

Multiple coagulation factor levels and hemostatic variables were determined in controls, vaccinated subjects and vaccinated subjects with a prior COVID-19 infection (Figure 2). Prothrombin and fibrinogen levels were significantly reduced after vaccination (−7.5%, *p* < 0.0001 and −16.9%, *p* < 0.0001), suggesting ongoing usage of coagulation factors. Many post-vaccination samples showed both prothrombin (42.1%) and fibrinogen levels (16.1%) outside of the normal range, which was significantly less in the control group (26.4% with *p* = 0.026, and 7.3% with *p* = 0.081, respectively; Figures 2A,C). FVIII levels were comparable between controls and vaccinated subjects (Figure 2B). Additionally, we found that 9% of the vaccinated subjects had D-dimer levels outside of the normal range, compared to none of the control subjects (*p* = 0.047).

### ChAdOx1-S vaccination and anticoagulant factor levels

Plasma antithrombin levels did not differ between controls, vaccinated subjects and vaccinated subjects with a prior COVID-19 infection. As for prothrombin and fibrinogen, the levels of antithrombin were outside of the normal range in 23.9% of the vaccinated individuals (compared to 3.6% in the control group (*p* = 0.001; Figure 2D). In contrast to the reduced fibrinogen levels, the plasma levels of another acute phase protein and thrombin inhibitor, α_2_M, increased significantly after vaccination in both the group without (+ 20.0%, *p* = 0.0008; Figure 2F) and the group with prior COVID-19 infection (+ 23.1%, *p* = 0.0006; Figure 2E).

### ChAdOx1-S vaccination and thrombin generation

**Figure 3:**
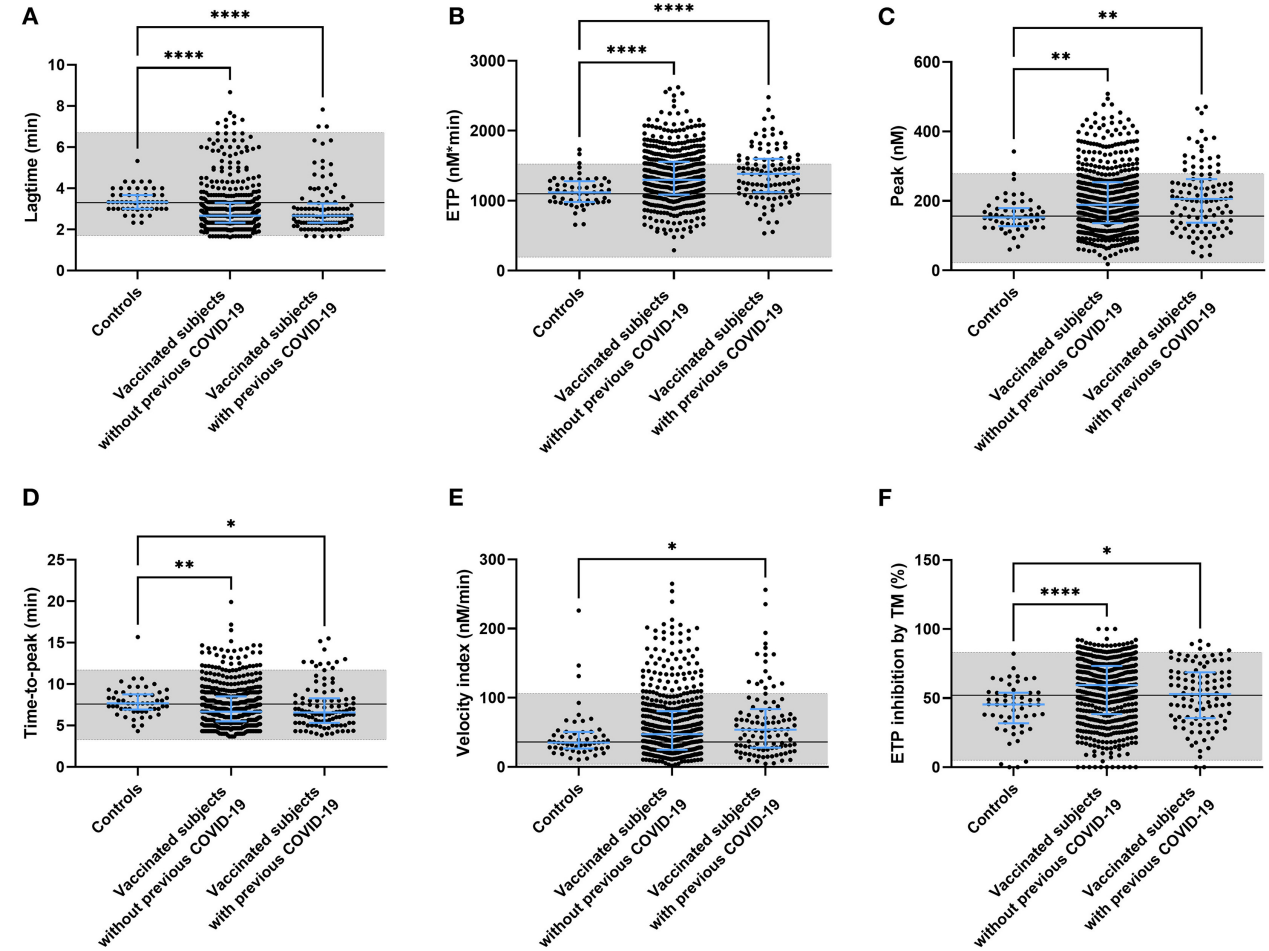
Thrombin generation parameters in controls and subjects vaccinated with ChAdOx1-S with and without prior COVID-19 infection. Thrombin generation was measured at 5 pM tissue factor and the lag time **(A)**, ETP **(B)**, peak height **(C)**, time-to-peak **(D)**, velocity index **(E)**, and the inhibition of the ETP by the addition of thrombomodulin **(F)** were quantified. Reference values for each parameter are indicated as the gray area in the dot plot. Differences between the groups were analyzed by ANOVA or Kruskal-Wallis analysis, depending on the distribution of the data. *P*-values below 0.05, 0.01, 0.001, and 0.0001 were marked as ^*^, ^**^, ^***^, and ^****^, respectively.

To better understand the effect of these changes in coagulation factor levels, we used the thrombin generation assay as an indicator of global coagulation. Figure 3 displays the thrombin generation parameters obtained after stimulation with 5 pM tissue factor (TF). Stimulation with 1 pM TF gave similar results. Both time-dependent parameters lag time and time-to-peak were significantly shorter in vaccinated subjects than controls (−12.7%, *p* < 0.0001 and −7.1% and *p* < 0.0001), as well as in vaccinated subjects with prior COVID-19 compared to control (−11.5%, *p* < 0.0001 and −7.3% and *p* = 0.0003) (Figures 3A,D). The endogenous thrombin potential, which is a marker of the overall clotting ability of a subject, was increased in vaccinated subjects without (+ 18.7%, *p* < 0.0001; Figure 3B) and with a history of COVID-19 (+ 22.4%, *p* < 0.0001). A similar effect was seen for the thrombin generation peak height, which was increased by 27.1% (*p* = 0.0034) and 32.6% (*p* = 0.0015) in vaccinated subjects with and without prior COVID-19 infection, respectively (Figures 3C,E). Additionally, we investigated the effect of the natural anticoagulant activated protein C system *via* the addition of thrombomodulin and the subsequent quantification of its inhibitory action on the endogenous thrombin potential (ETP) (Figure 3F). Vaccinated subjects had a significantly larger response to the addition of thrombomodulin, regardless of their history of COVID-19 infection.

### ChAdOx1-S vaccination and von willebrand factor

**Figure 4:**
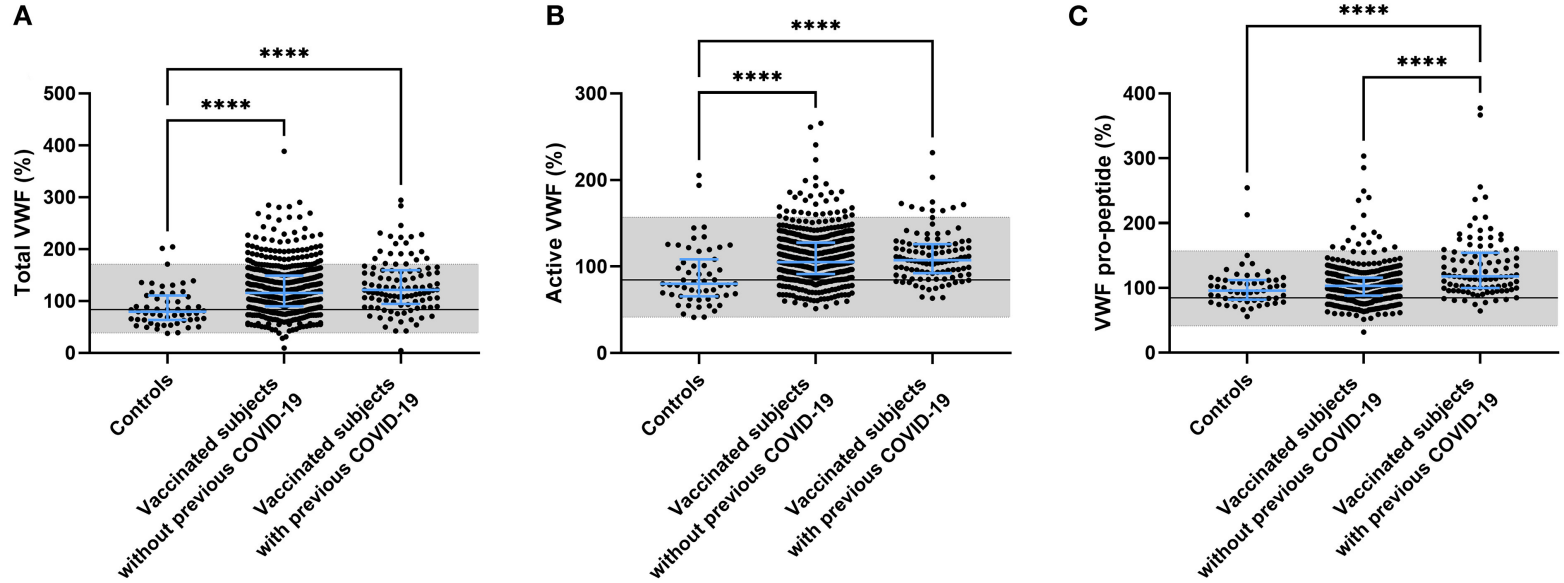
Endothelial activation in controls and subjects vaccinated with ChAdOx1-S with and without prior COVID-19 infection. Endothelial activation was assessed by the measurement of total VWF **(A)**, active VWF **(B)** and VWF pro-peptide **(C)**. Reference values for each test are indicated as the gray area in the dot plot. Differences between the groups were analyzed by ANOVA or Kruskal-Wallis analysis, depending on the distribution of the data. *P*-values below 0.05, 0.01, 0.001, and 0.0001 were marked respectively.

Another component that is known to play a role in the pathogenesis of thrombosis is the vessel wall. An important biomarker of endothelial activation is von Willebrand factor (VWF). We quantified the amount of circulating total VWF, active VWF (VWF in a GPIb-binding conformation) and VWF pro-peptide in controls and vaccinated subjects. Both total and active VWF were significantly higher in vaccinated subjects (+ 39.5%, *p* < 0.0001 for total VWF and +24.1 %, *p* < 0.0001 for active VWF) and this effect seemed to be more pronounced in vaccinated subjects with a prior COVID-19 infection (+ 47.8%, *p* < 0.0001 for total VWF and +26.2 %, *p* < 0.0001 for active VWF; Figures 4A,B) although it did not reach significance. We did find that VWF pro-peptide was significantly increased in subjects with a history of COVID-19 compared to controls (+ 30.7%, *p* < 0.0001) and compared to vaccinated subjects without prior COVID-19 (+27.8%, *p* < 0.0001) (Figure 4C).

### The association of anti-SARS-CoV-2 spike protein antibody titer and hemostatic parameters

**Figure 5:**
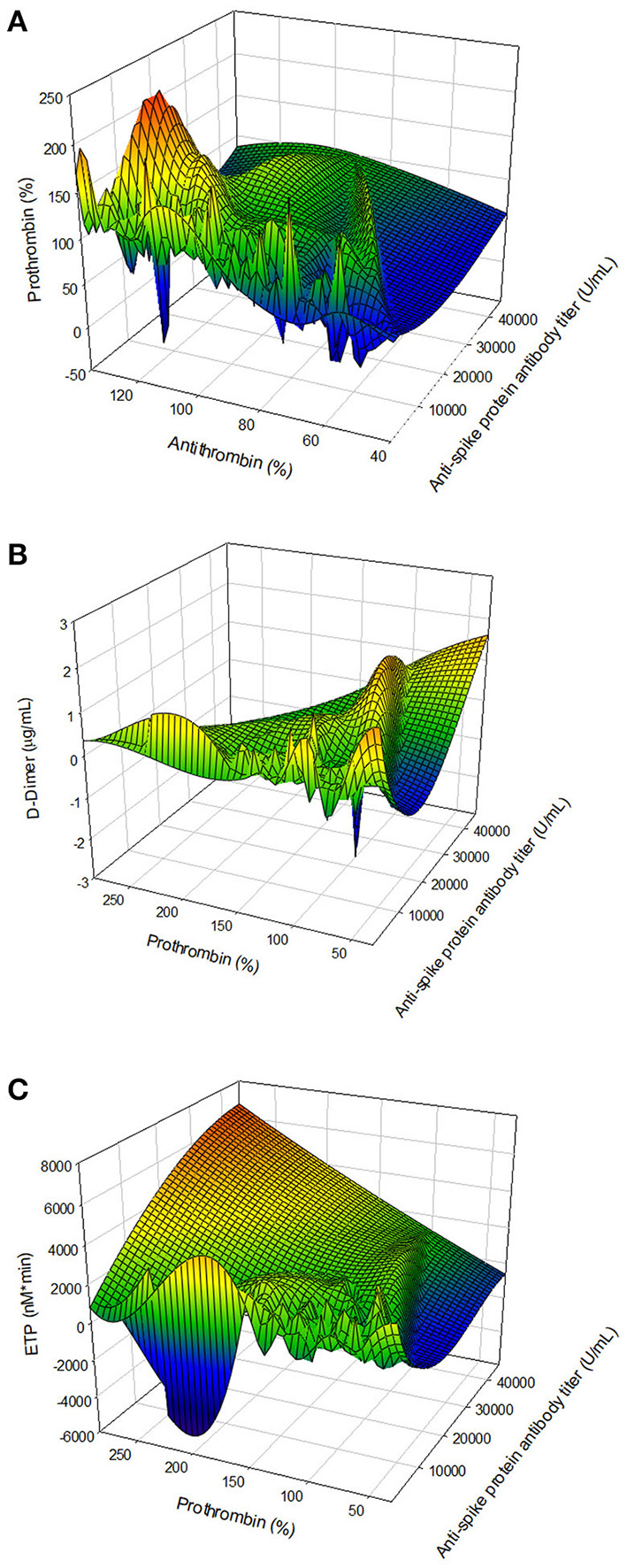
The association of the anti-SARS-CoV-2 spike protein antibody titer and hemostatic parameters. **(A)** The relationship antibody titer and coagulation factor levels: antithrombin and prothrombin. **(B)** The association of antibody titer and coagulation factor consumption: prothrombin and D-dimer. **(C)** The relation of antibody titer and the thrombin generation potential, quantified by the ETP and prothrombin level.

We observed that vaccination with ChAdOx1-S affected several parts of the hemostatic system. To further investigate this, we studied a possible relationship with the titer of the anti-SARS-CoV-2 spike protein antibody titer with hemostatic parameters. Figures 5A–C shows that a higher anti-SARS-CoV-2 spike protein antibody titer is associated with a lower prothrombin and antithrombin level. Additionally, in subjects with a high anti-spike protein titer, D-dimer levels were higher when prothrombin levels were low. Assessing thrombin generation, the endogenous thrombin potential is higher in subjects with a high anti-spike protein titer compared to subjects with a low titer, at comparable prothrombin levels.

### ChAdOx1-S vaccination and interleukin levels

Furthermore, in the pathogenesis of COVID-19 infection, IL-6 and IL-10 have been implicated as a link between the immune system and coagulation. We investigated the role of the immune system in the apparent haemostatic changes after vaccination with ChAdOx1-S by measuring interleukin (IL) 6 and IL-10 in a subset of the population. IL-6 was comparable between the vaccinated population and the control group (data not shown). On the contrary, IL-10 was significantly higher in the group of vaccinated subjects, compared to control subjects (9.29 pg/mL ± 16.9 pg/mL vs. 2.43 pg/mL ± 3.58 pg/mL, *p* = 0.032; data not shown).

### The association of post-ChAdOx1-S vaccination clinical symptoms and hemostatic parameters

**Figure 6:**
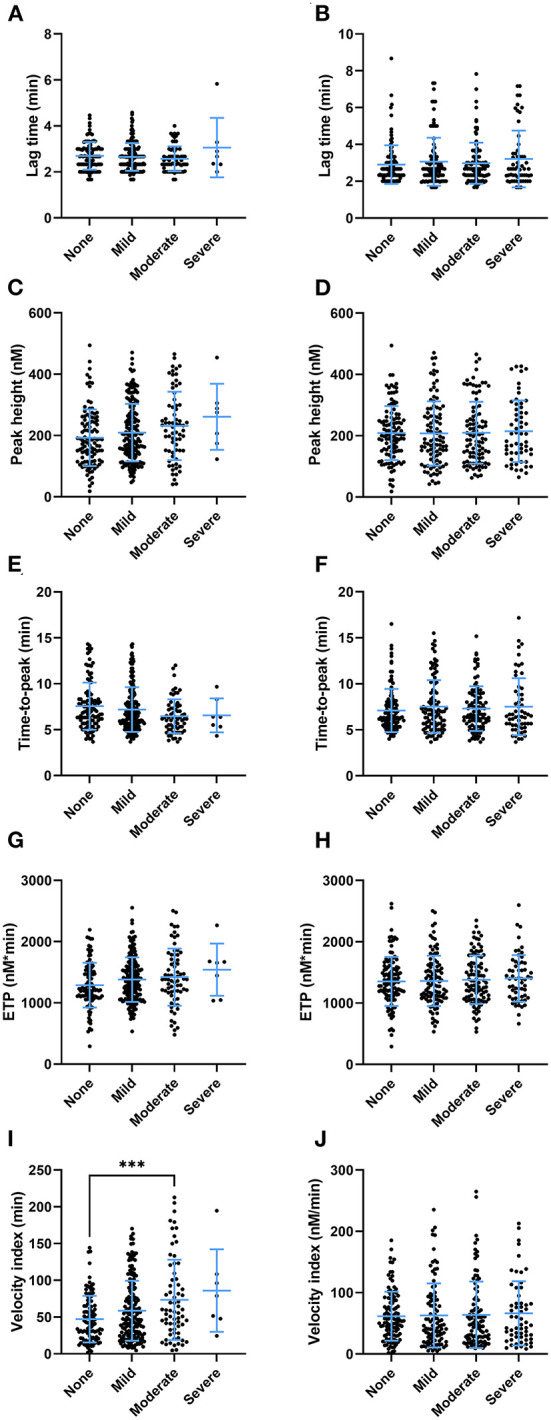
The association of thrombin generation parameters and clinical symptoms after the second round of vaccination as reported by subjects vaccinated with ChAdOx1-S with and without prior COVID-19 infection. **(A,B)** The lag time did not differ significantly at the increasing levels of injection site symptoms **(A)** or systemic symptoms **(B)**. The peak height differed significantly between subjects with increasing injection site symptoms (*p* < 0.038), although the study was underpowered to detect differences between specific groups in *post hoc* analysis **(C)**. Increasing systemic symptoms did not result in differences in peak height **(D)**. **(E,F)** The time-to-peak differed significantly between the categories of injection site symptoms (*p* = 0.029) **(E)**, but not systemic symptoms **(F)**. **(G,H)** The ETP was not significantly affected by the various levels of injection site symptoms **(G)** or systemic symptoms **(H)**. **(I)** The velocity index was significantly increased in subjects with more injection site symptoms (*p* < 0.001) **(I)**, but not systemic symptoms **(J)**. Data are displayed as mean and standard deviation or as percentages in the case of categorical variables. Differences between the groups were analyzed by ANOVA or Kruskal-Wallis analysis, depending on the distribution of the data. A *p*-value below 0.05 was considered statistically significant and ^***^ indicates a *p*-value below 0.001.

Clinical symptoms after the second round of vaccination were recorded. We investigated whether there was an association between clinical adverse effects related to the vaccination with coagulation factor levels (Table 2) and thrombin generation parameters (Figure 6). Prothrombin levels were significantly lower (100% ± 19% vs. 104% ± 19%, *p* = 0.027) in subjects that reported injection site symptoms compared to subjects that did not report injection site symptoms. Antithrombin levels were lower as well in subjects that reported injection site symptoms (95% ± 18% vs. 101% ± 19%, *p* = 0.005). Fibrinogen, α_2_Macroglobulin, FVIII, and D-dimer levels were comparable between vaccinated subjects with and without injection site symptoms. Moreover, whereas total VWF and active VWF levels were the same in subjects with and without injection site symptoms, VWF pro-peptide levels were 13% lower in subjects with injection site symptoms (*p* < 0.001). Subjects that reported systemic symptoms had significantly lower FVIII levels compared to subjects that did not report systemic symptoms (117% ± 33% vs. 127% ± 41%, *p* = 0.027) Interestingly, increasing severity of injection site symptoms (Figures 6A,C,E,G,I), but not systemic symptoms (Figures 6B,D,F,H,J), is associated with an increasingly more procoagulant thrombin generation profile (Figure 6). This is reflected by a higher peak height (+ 8.3, + 19.7, and +35.2% in subjects with mild, moderate and severe injection site symptoms compared to symptom free subjects (*p* = 0.038) (Figure 6C). Moreover, the time-to-peak shortened with increasing symptoms severity (*p* = 0.028) (Figure 6E), and the velocity index increased from 47 nM/min ± 32 nM/min in symptoms free subjects, to 59 nM/min ± 41 nM/min in subjects with mild symptoms, 73 nM/min ± 55 nM/min in subjects with moderate symptoms and 89 nM/min ± 56 nM/min in subjects with severe symptoms (*p* = 0.0003) (Figure 6I).

**Table 2:** The association of coagulation factor levels and clinical symptoms after the second round of vaccination as reported by subjects vaccinated with ChAdOx1-S with and without prior COVID-19 infection.

**Coagulation factor**	**Injection site symptoms**			**Systemic symptoms**		
	*No Symptoms*	*Symptoms*	*P-value*	*No Symptoms*	*Symptoms*	*P-value*
Prothrombin, % (SD)	104 (19)	100 (19)	*0.027*	103 (19)	100 (19)	*n.s*.
Antithrombin, % (SD)	101 (19)	95 (18)	*0.005*	98 (20)	97 (19)	*n.s*.
Fibrinogen, g/L (SD)	2.60 (0.81)	2.51 (0.84)	*n.s*.	2.59 (0.86)	2.54 (0.84)	*n.s*.
α2-Macroglobulin, μM (SD)	3.97 (1.26)	4.15 (1.27)	*n.s*.	3.98 (1.18)	4.14 (1.30)	*n.s*.
FVIII, % (SD)	126 (42)	120 (38)	*n.s*.	127 (41)	117 (33)	*0.027*
D-Dimer, μg/mL (SD)	0.30 (0.04)	0.30 (0.04)	*n.s*.	0.30 (0.04)	0.30 (0.04)	*n.s*.
VWF, % (SD)	125 (52)	125 (45)	*n.s*.	128 (45)	120 (41)	*n.s*.
active VWF, % (SD)	112 (26)	112 (27)	*n.s*.	114 (26)	111 (27)	*n.s*.
VWF pro-peptide, % (SD)	131 (45)	114 (33)	* <0.001*	125 (43)	117 (35)	*n.s*.

Data are displayed as mean and standard deviation or as percentages in the case of categorical variables. Differences between the groups were analyzed by the student T-test or the Mann Whitney test, depending on the distribution of the data. A p-value below 0.05 was considered statistically significant.

## Discussion

Vaccination against COVID-19 has been adopted all over the world with a few exceptions. It has been shown that in general vaccination with either one of the four vaccines approved by the FDA/EMA - ChAdOx1-S (Vaxzevria; Astra Zeneca), Ad26.COV2-S (Janssen COVID-19 Vaccine; Johnson and Johnson) and two COVID-19 mRNA Vaccines (Comirnaty and Spikevax, respectively from Pfizer and Moderna), is safe (13–16). Despite the acceptable safety records, rare incidences of thrombosis after vaccination of which VITT in relation to COVID-19 vaccination have reached the news worldwide. Interestingly, and less well-known, a large Scandinavian study revealed that the risk of venous thromboembolic events is almost double in subjects after receiving the ChAdOx1-S vaccine, as 59 events occurred in their cohort (*N* = 281,264), while 30 events were expected (17).

In our study we investigated possible changes in haemostasis after full vaccination with ChAdOx1-S related to an increased risk of thrombosis. We studied 631 individuals after receiving ChAdOx1-S and found three clusters of changes: (1) Lower coagulations levels indicating usage of coagulation factors; (2) Increased and faster thrombin generation indicating a more active coagulation system; and (3) increased VWF levels indicating activation of the vascular wall. All three clusters together show that the haemostatic system is shifted to a more procoagulant state compared to unvaccinated controls.

Several mechanisms may explain these changes at 4 weeks after vaccination. First, vaccination triggers antibody formation and our results indicate the thrombin generation profile becomes more procoagulant in subjects vaccinated with ChAdOx1-S, shown by the increased endogenous thrombin potential both in vaccinated subjects with or without prior COVID-19 infection. Other groups have shown that ChAdOx1-S vaccination causes a transient increase in TG (18), although they could not detect a significant difference between vaccination subjects and unvaccinated controls (19). Moreover, our results show that the endogenous thrombin potential is correlated with the magnitude of anti-spike protein antibody titer. The more procoagulant TG profile is further reflected in an increased TG peak height in vaccinated subjects and shortened time-dependent TG parameters, which indicates that the TG potential is not only increased, but that TG is also triggered faster.

We did not find a direct cause of the increase of the thrombin generation profile, although significant associations exist between the TG parameters and the titer of the anti-spike protein antibodies. Coagulation factors with established procoagulant effects such as prothrombin, FVIII and fibrinogen are not increased or even decreased upon vaccination with ChAdOx1-S (12, 20–22). Moreover, vaccinated subjects are more sensitive to the anticoagulant effect of thrombomodulin (TM) than controls, and therefore, the changes found in TG cannot be attributed to reduced activation of the activated protein C pathway. Nevertheless, the reduction of procoagulant factor levels in combination with an increased D-dimer and an increased thrombin generation potential, point toward a mild consumption of coagulation factors *in vivo* due to ongoing activation of the coagulation system.

Second, our results show that vaccination induces endothelial activation, as reflected by an increase of total VWF and active VWF in the blood stream, and the elevated levels of VWF pro-peptide indicate that this is a recent effect in the post-vaccination period. In severe SARS-CoV-2 infections, the vascular endothelium is the cornerstone of organ dysfunction and recent data have emphasized the crucial role of endothelial cells in vascular dysfunction, immune-thrombosis and inflammation (23, 24). Moreover, vascular activation is a strong predictor of mortality in COVID-19 disease on the ICU (25). Post COVID-19 infection endothelial activation is still measurable at 3 months after hospital discharge (17, 26) and may comprise part of the long COVID syndrome (27). It remains unknown which mechanisms trigger late endothelial activation following vaccination, mimicking elements of COVID-19.

Third, others have shown that single cell sequencing of peripheral blood mononuclear cells before and at 28 days after vaccination with an inactivated SARS-CoV-2 vaccine showed consistent changes in gene expression levels in many different immune cell types, also including inflammatory pathways regulated by the NFkB system. These changes, reflecting the cellular immune response to vaccination, were associated with, amongst others, detectable alterations in inflammatory mediators and global coagulation tests, suggesting that immune cell priming could also have protracted effects on hemostasis, maybe unrelated to antibody responses (28). One of the main procoagulant drivers, tissue factor, is regulated through the NFkB system, which is amplified in the study by Liu and colleagues, suggesting that monocytic, or extracellular vesicle mediated expression of tissue factor, may be an additional trigger of thrombin generation observed in our study. The fact that the key cytokine Il-6, that shows an early small peak 1 day after vaccination with ChAdOx1-S (26), is no longer elevated at 4 weeks, makes it less likely that the procoagulant changes in this study are the direct consequence of pro-inflammatory cytokine release. However, alike the IL-10 increase in patients with a COVID-19 infection, IL-10 was found to be elevated as well in vaccinated subjects, regardless of the history of COVID-19 infection in the past. IL-10 is known to attenuate coagulation and fibrinolysis (29). Strangely enough, the role of IL-10 is not fully known and somewhat ambiguous in COVID-19. As we found increased IL-10 levels, this further indicates that the immune system is still active, which might also contribute to the prolonged hemostatic changes that we have found in the vaccinated population.

A limitation of this study is that it mainly studies the effect of ChAdOx1-S vaccination in women, as the predominant part of the healthy workers are female. Subsequently, an unvaccinated pre-COVID-19 control group was chose for result comparison. These samples were stored longer at −80°C than the samples of vaccinated subjects, because it was difficult at the time of the study to include controls that were unvaccinated and naïve to the SARS-CoV-2 virus. Sex is known to affect thrombin generation and coagulation factor levels and function. Therefore, we included reference values obtained in a more heterogenous group of 120 healthy adults (50% men, 37% women without oral contraceptives and 13% women with oral contraceptives) for each parameter measured in this study. Moreover, due to the large group size, some results are statistically significant, although the clinical significance of small changes in these parameters might be debatable. Additionally, there was were not much data available on the background of the vaccinated subjects and unvaccinated controls, such as body mass index, smoking status, or oral contraceptive use.

The net impression of our findings is that 4 weeks after vaccination with ChAdOx1-S, a substantial subset of individuals still show signs of endothelial and hemostatic activation that may explain at least part of the observed apparent increase in thromboembolic events after vaccination. Whether these effects are specific for this vaccine and would also appear to some extent after vaccination with Ad26.COV2-S or even the mRNA vaccines, remains to be determined. VITT is a very rare event occurring earlier on after vaccination and although hemostatic changes like the ones we observed could theoretically contribute to a subject's sensitivity to develop immune thrombosis, this conclusion cannot be drawn from the present data. Other rare thromboembolic events, apparently unrelated to VITT (eg without thrombocytopenia) have also been noted after vaccination (17, 30) and presumably, the individual variation in immune and procoagulant responses, could contribute to the risk of thrombosis.

It is obvious from the cumulative literature data that the risks of COVID-19 by far exceed the very small risks of (immune) thrombosis, whether VITT or not, and our data should in no way be used to rally against the benefits of vaccination. Nevertheless, the mechanistic data from this study underline how closely immunity and hemostatic systems are intertwined, illustrating the evolutionary importance of both systems in defense against invading pathogens.

## Data availability statement

The original contributions presented in the study are included in the article/supplementary materials, further inquiries can be directed to the corresponding author.

## Ethics statement

The studies involving human participants were reviewed and approved by Comite Medische Ethiek Ziekenhuis Oost-Limburg. The patients/participants provided their written informed consent to participate in this study.

## Author contributions

BL, LH, RH, HC, and DS supervised the study. MN, MR, DH, and HS performed experiments and analyzed the data. DM, ST, KV, JP, PV, RD, ML, and TF collected patient samples, processed the data and critically read the manuscript. RdL-K analyzed the data and prepared the figures. BL and RdL-K drafted the manuscript. HS, MN, RH, LH, HC, and DS critically revised it. All authors contributed to the article and approved the submitted version.

## Conflict of interest

BL, RdL-K, MR, DH, and MN are employees of Synapse Research Institute, part of Diagnostica Stago. HC received funding for research from Bayer and Pfizer; compensation fees for consultancy and advisory boards from Daaichi, Pfizer, Leo, Bayer, Galapagos, Anthos, Alexion, and Alveron; shareholder from Coagulation profile; all benefits were transferred to the CARIM institute to support investigator-initiated research. The remaining authors declare that the research was conducted in the absence of any commercial or financial relationships that could be construed as a potential conflict of interest.

## Publisher's note

All claims expressed in this article are solely those of the authors and do not necessarily represent those of their affiliated organizations, or those of the publisher, the editors and the reviewers. Any product that may be evaluated in this article, or claim that may be made by its manufacturer, is not guaranteed or endorsed by the publisher.
